# Pulmonary Artery Stenosis After an Orthotopic Heart Transplantation: A Case Report With Cardiac Imaging Findings and a Literature Review

**DOI:** 10.7759/cureus.57416

**Published:** 2024-04-01

**Authors:** Pritish Aher, Nini Saad, Aman Aher, Sarv Priya, Alessandra Albini

**Affiliations:** 1 Radiology, University of Miami Miller School of Medicine, Jackson Memorial Hospital, Miami, USA; 2 Nutrition and Exercise Physiology, University of Missouri, Columbia, USA; 3 Radiology, University of Iowa Hospitals and Clinics, Iowa City, USA

**Keywords:** mr pulmonary angiography, pulmonary artery velocity, pulmonary artery hypertension, cardiac magnetic resonance imaging, pulmonary artery stenosis

## Abstract

Pulmonary artery stenosis is a rare complication of heart transplantation. It is typically a congenital condition or can be secondary to rheumatic fever, systemic vasculitis like Behcet’s disease, or Takayasu’s arteritis. It can also occur as a rarity of a delayed complication post-heart transplant. In this report, we describe the imaging findings of pulmonary artery stenosis in a patient who underwent an orthotopic heart transplant more than 10 years prior. Dynamic cardiac magnetic resonance imaging (MRI), phase contrast imaging, and MR angiography in the management of pulmonary artery stenosis helped in heart and pulmonary circulation. Functional evaluation can be achieved with current multichannel transmit-receive coils. Cardiac gated pre- and dynamic contrast-enhanced MR was performed with phase-contrast imaging for further evaluation confirming the diagnosis of pulmonary artery stenosis.

## Introduction

Pulmonary artery stenosis, which occurs due to narrowing of the pulmonary artery anastomotic site, restricts blood flow between the heart and lungs. The associated increase in pressure gradient can cause right heart dysfunction, possibly leading to right heart failure. Patients may experience shortness of breath, dyspnea, chest pain and limited exercise capacity. Pulmonary artery stenosis is typically congenital or can be secondary to rheumatic fever, systemic vasculitis like Behçet’s disease, or Takayasu’s arteritis [1]. It can also occur as a rare complication of heart transplantation years after transplantation. The cause of pulmonary artery stenosis after heart transplantation is unknown.

Echocardiography is often the first imaging modality employed as it offers functional assessment but may be limited by other mediastinal structures and artifacts [2]. Cardiac catheterization can assess pressure gradient and valve function, while MRI and CT scans offer better visualization of anatomic location and secondary changes [3]. Treatment may involve lifestyle changes, medications, and surgery depending on the clinical findings, patient age, and comorbidities.

We report imaging findings of pulmonic stenosis presenting 12 years after an orthotopic heart transplantation.

## Case presentation

In this report, we describe the imaging findings of a 50-year-old male patient who presented with pulmonary artery stenosis more than 10 years after receiving an orthotopic heart transplantation. The patient has a history of idiopathic dilated cardiomyopathy, hypertension, mixed hyperlipidemia, anemia, neuropathy, and pulmonary hypertension with elevated pulmonary artery pressures. The orthotopic heart transplantation complicated with large pericardial effusion was treated with successful pericardiocentesis. The patient was on chronic immunosuppression and has had neuropathy & myopathy with Tacrolimus use and was transitioned to Sirolimus. The patient has regularly followed up with cardiology and transthoracic echocardiography.

A recent transthoracic echocardiogram demonstrated a flatted septum and persistently dilated inferior vena cava consistent with right heart strain. The left and right atrium were enlarged. The right ventricle was mild to moderately enlarge with mild-moderate mitral and tricuspid regurgitation. The patient had cardiac catheterization with elevated pulmonary artery pressure of 48/21mm of Hg, right ventricular pressure of 55/12 mm of Hg, and right atrial pressure of 12 mm of Hg. The findings were consistent with pulmonary hypertension. Pulmonary hypertension is defined as an increase in the mean pulmonary arterial pressure (≥25 mmHg) at rest. With elevated pulmonary artery pressure, a cardiac gated pre- and dynamic contrast-enhanced magnetic resonance imaging (MRI) was performed with phase contrast imaging for further evaluation. With cardiac MRI, we identified the pulmonary artery stenotic segment and flow velocity changes confirming the diagnosis of pulmonary artery stenosis. With cardiology follow-up, the patient's pulmonary hypertension was medically managed.

**Figure 1:**
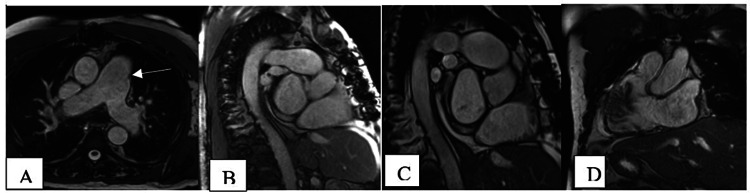
Cardiac MRI. A: Axial T2 images shows dilated main pulmonary artery with narrowing of main pulmonary artery atanastomotic site (White arrow). B and C: Short axis TRUFI images shows short segment narrowing of main pulmonary artery. D: TRUFI RVOT image shows dilated main pulmonary artery

**Figure 2:**
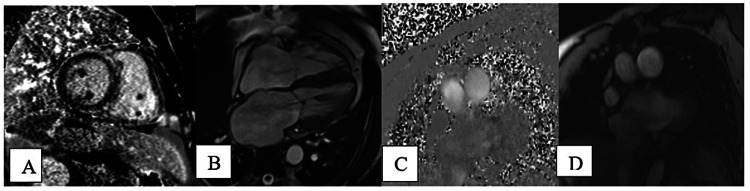
Cardiac MRI with post gadolinium and phase contrast images. A: Post-gadolinium short axis image shows RV insertion site enhancement. B: Four-chamber SSFP image shows right ventricular dilatation and thickening of the right ventricular wall. C and D: Phase and magnitude images at the level of the prestenotic pulmonary artery.

Cardiac gated pre- and dynamic contrast-enhanced MR was recommended for RV pressure and volume overload. The cardiac MRI was performed with a 1.5 Tesla magnet and showed a left ventricle ejection fraction of 56%, right heart strain with septal dyskinesia, and post-ductal segment pulmonary artery stenosis. Focal stenosis was observed in the distal main pulmonary artery with a lumen of 2.1 cm; the dilated proximal main pulmonary artery measured 4.3 cm and the distal artery measured 2.9 cm (Figure 1A-D). No adjacent soft tissue pathology was seen. Moderate mitral regurgitation and mild tricuspid regurgitation, with dilated right ventricle, right atrium, and left atrium were noted. Phase-contrast imaging demonstrated elevated pulmonary artery flow velocities measuring 38 cm/s at the pre-stenotic main pulmonary artery, 58 cm/s at the post-stenotic main pulmonary artery, 54 cm/s at the right pulmonary artery, and 34 cm/s at the left pulmonary artery, consistent with pulmonary artery stenosis and pulmonary artery hypertension. No post-stenotic dilatation of the pulmonary artery was observed. The T1 and T2 mapping values were above two standard deviations from the mean at the anteroseptal and inferoseptal right ventricle insertion site, measuring 1262 and 66 respectively. The borderline thickening of the right ventricular wall was 5.2 mm (Figures 2A-D). Resting perfusion images demonstrated no evidence of a defect, and delayed enhancement images demonstrated focal areas of delayed enhancement of the anterior and interior RV insertion sites. The patient is still being worked up by cardiology.

After four months of medical management, repeat cardiac catheterization showed that the pulmonary artery pressure was reduced to 30/12 mm of Hg, right ventricular pressure to 30/6 mm of Hg, and right atrial pressure to 6 mm of Hg.

## Discussion

This case highlights the imaging findings of the delayed complication of pulmonary artery stenosis in a patient who underwent orthotopic heart transplantation more than 10 years ago.

Heart transplantation is the treatment for patients with end-stage heart failure (HF) refractory to medical and resynchronization therapy. Amongst the orthotopic and heterotropic heart transplants, orthotopic heart transplantation is more common. In this method, the heart from the deceased donor is placed into the recipient's pericardial sac right after the removal of the recipient's diseased heart. This method has two fundamental approaches: the biatrial method, and the bicaval method. In the biatrial method, the recipient's heart is extracted along with a segment of the native atria, known as the atrial cuff, which remains in place to be connected to the donor heart. Consequently, there are only four connections: two atria, the aorta, and the pulmonary trunk. However, the suture line along the atrial cuff is linked to a heightened occurrence of arrhythmias. This approach is the initial and swiftest procedure. In the bicaval method, the recipient's heart is excised along with the native atria, facilitating multiple anastomoses: the right atrium to the superior vena cava (SVC) and inferior vena cava (IVC), and the left atrium to the pulmonary veins, aortic anastomosis, and pulmonary trunk anastomosis. Consequently, this approach involves more connections and requires a longer procedure duration compared to the biatrial method. However, as the natural anatomy of the atria is preserved, there are fewer complications related to the sinoatrial (SA) node [3,4].

Pulmonary artery stenosis can occur anywhere along the main pulmonary artery, or the right or left pulmonary artery. The majority of cases are congenital and present in the pediatric age group. Pulmonary artery stenosis in adults is typically seen with severe cardiac structural diseases, rheumatic heart disease, systemic vasculitis, cardiac tumors, or previous cardiothoracic surgeries [1,2]. Most cases of pulmonary artery stenosis are mild and present without symptoms. Symptomatic patients typically present with progressive dyspnea on exertion or fatigue, and in severe cases, patients may experience angina or sudden cardiac arrest. Pulmonary artery stenosis progressively causes increased systolic pressure in the right ventricle, right ventricular hypertrophy, and right ventricular dilation, followed by a decrease in cardiac output [5,6].

The initial evaluation of pulmonary artery stenosis and pulmonary hypertension is invariably by echocardiography, as it can assess the pulmonary valve, jet velocity, pressure gradients, and the right-sided chambers of the heart. However, echocardiography has a limitation in anatomic and hemodynamic evaluation of pulmonary artery stenosis [1]. For the diagnosis of pulmonary artery stenosis, cardiac catheterization and pulmonary angiography are required. Cardiac magnetic resonance imaging (CMR), MR-phase contrast imaging (PC), and MR pulmonary angiography are an alternative to echocardiography and supplement the findings of cardiac catheterization, especially in the setting of suboptimal echocardiographic exam, or complicated anatomy [7]. CMR also provides a noninvasive measurement of the right ventricular volume, function, and pulmonary artery hemodynamics. Cross-sectional MRI imaging can provide greater anatomic detail like vessel diameter at the stenotic segment, pre- and post-stenotic pulmonary artery diameter, localization of stenosis, and length of stenosis. With 2D PC techniques, velocities are measured through an acquisition plane. With four-dimensional PC imaging, a complete time-resolved, three-directional blood-flow velocity field in a volume can be assessed. With 2D PC technique, peak flow velocities are measured at the main, right, and left pulmonary arteries separately. Similarly, with 2D PC-MRI technique, the gradient at the stenotic segment is measured with peak pulmonary flow velocity at the pre-stenotic and post-stenotic segments. With the gradient, the degree of stenosis is suggested. Low pulmonary artery flow velocity is indicative of pulmonary hypertension [8]. With multichannel transmit-receive coils, cardiac MRI and MR angiography have emerged as an important diagnostic tool in the management of pulmonary arterial hypertension and pulmonary artery stenosis, as this allows dynamic imaging of both the heart and pulmonary circulation. Mapping of pulmonary arteries at 3D MR angiography can accurately localize and characterize the stenosis. MR imaging, especially, is useful in the postoperative evaluation of pulmonary valve function where anatomic changes and postoperative hardware can create significant challenges when using echocardiography [9,10]. If patients cannot undergo CMR, then cardiac computed tomography (CCT) may be advised [10]. 

Those with isolated pulmonary artery stenosis will require ongoing cardiac monitoring to evaluate the progression of stenosis, right ventricular hypertrophy, heart failure, or arrhythmias, such as premature atrial contractions, premature ventricular contractions, and ventricular couplets [6]. The prognosis for most patients with pulmonary artery stenosis is excellent but may require further interventions later.

On histopathology, the stenotic segment shows intimal proliferation with marked medial thickening, and increased and disorganized elastic fibers. Patients with chronic vasculitis show chronic inflammatory changes along with microthromboembolism [2].

## Conclusions

Pulmonary artery stenosis, typically a congenital condition, may occur as a rare complication of heart transplantation. Very little is known about how pulmonary artery stenosis develops after a heart transplantation. In this report, we describe the imaging findings of a patient who presents with pulmonary artery stenosis 12 years after receiving an orthotopic heart transplantation. Given the rarity of this complication, imaging findings may provide insight into this rare complication. Due to the dynamic imaging of both the heart and pulmonary circulation, a cardiac MRI, an MRI phase contrast study, and MR pulmonary angiography are important diagnostic modalities in the management of pulmonary artery stenosis. Mapping of pulmonary arteries at 3D MR angiography can accurately localize and characterize the stenosis. In addition, MR is excellent at evaluating peak velocities, valvular regurgitation, and other hemodynamics. 
